# From novel targets to potential therapies: development of antisense oligonucleotides for progressive supranuclear palsy

**DOI:** 10.1002/alz70859_100315

**Published:** 2025-12-25

**Authors:** Yuhao Min, Jeremiah Bergman, Jianna Tan, Xue Wang, Katie D. Sotelo, Joseph S. Reddy, Zachary Quicksall, Thuy T. Nguyen, Adriana O Mitchell, Dennis W. Dickson, Keiji Kawatani, Grace A. Selecky, Kristen R. Whitney, Pablo Martinez, Cristian A Lasagna Reeves, John F. Crary, Takahisa Kanekiyo, Mariet Allen, Nilüfer Ertekin‐Taner

**Affiliations:** ^1^ Mayo Clinic, Jacksonville, FL USA; ^2^ Ronald M. Loeb Center for Alzheimer's Disease, New York, NY USA; ^3^ Icahn School of Medicine at Mount Sinai, New York, CA USA; ^4^ Icahn School of Medicine at Mount Sinai, New York, NY USA; ^5^ Friedman Brain Institute, New York, NY USA; ^6^ Indiana University School of Medicine, Indianapolis, IN USA; ^7^ Stark Neurosciences Research Institute, Indiana University School of Medicine, Indianapolis, IN USA; ^8^ Department of Anatomy, Cell Biology, and Physiology, Indiana University School of Medicine, Indianapolis, IN USA; ^9^ Center for Computational Biology and Bioinformatics, Indiana University School of Medicine, Indianapolis, IN USA; ^10^ Department of Artificial Intelligence & Human Health, Nash Family Department of Neuroscience, Ronald M. Loeb Center for Alzheimer's Disease, Friedman Brain Institute, Neuropathology Brain Bank & Research CoRE, Icahn School of Medicine at Mount Sinai, New York, NY USA

## Abstract

**Background:**

Progressive supranuclear palsy (PSP) is a devastating neurodegenerative disorder with rapid progression to death. Multiple efforts to develop a disease‐modifying therapy for PSP have yet to succeed. We previously reported the discovery of three potential therapeutic targets for PSP. These three genes, DDR2, KANK2, and STOM, are consistently upregulated in the brains of PSP patients and mouse models of tauopathy. Reducing their expression in a drosophila model significantly rescued the tau‐induced eye degeneration and tau seeding activity. To translate these targets into the clinic, we are developing antisense oligonucleotides (ASO) as potential therapies.

**Method:**

We screened candidate ASOs in neuroglioma cell line H4. ASO hits were validated for their potency and safety. We also used iPSC‐derived neurons from PSP patients to test the potency and potential toxicity of our ASOs. To investigate target engagement, therapeutic efficacy and preliminary pharmacodynamic properties, we treated iPSC‐derived midbrain organoids with the ASO leads. Lastly, we will assess the off‐target effects of the ASOs by investigating the levels of mRNA transcripts that share similar sequences as our targets using RNAseq.

**Result:**

Our in vitro screening identified ASO leads that significantly reduce the target gene expressions at mRNA and protein levels in a dose‐dependent manner. The ASO hits also exhibit low cellular toxicity in the H4 cell line. Upon treating iPSC neurons with ASO hits, we were able to validate the dose‐dependent reduction of target mRNAs. Cell viability analysis indicated low toxicity, suggesting that these ASOs are potent and safe. In midbrain organoid model, DDR2‐targeting ASO showed time‐dependent target engagement and were prioritized for further development.

**Conclusion:**

We developed candidate therapeutics for PSP using ASOs that silence novel therapeutic targets discovered in our systems based cross‐species screening. We showed that our ASOs have low toxicity and confirmed target engagement at both mRNA and protein levels in vitro. The results suggest a favorable preclinical profile of our ASO leads that warrants additional investigations. Importantly, PSP shares congruent pathophysiological changes with other tauopathies, such as Alzheimer’s disease. These common perturbed pathways indicate that our ASOs can be potentially repurposed for multiple neurodegenerative tauopathies.

